# Community Rates of Internet Use and Cognitive Challenges Among Older Adults in MA & RI

**DOI:** 10.1093/geroni/igaf122.2558

**Published:** 2025-12-31

**Authors:** Dongfang Hong, Mengshi Liu, Sophia Casale, Calvin Tran, Elizabeth Dugan

**Affiliations:** University of Massachusetts Boston, Boston, Massachusetts, United States; University of Massachusetts Boston, Boston, Massachusetts, United States; University of Massachusetts Boston, Boston, Massachusetts, United States; University of Massachusetts Boston, Boston, Massachusetts, United States; University of Massachusetts Boston, Boston, Massachusetts, United States

## Abstract

Cognitive health is vital for aging well. Internet use may support cognitive health through social engagement and mental stimulation. This study examines the relationship between internet use and cognitive difficulties among older adults (65+) in Massachusetts and Rhode Island, highlighting potential digital inequalities. This study utilizes data from the 2025 Healthy Aging Reports (see healthyagingdatareports.org). Specific data sources: internet use 60 + (BRFSS 2018-2022), Alzheimer’s disease prevalence age 65 + (CMS 2020-2021), and self-reported cognition difficulty age 65 + (ACS 2018-2022). Small area estimation techniques were used to calculate age and sex-adjusted rates at the community and state level. Results show that in MA, 70.57% of individuals aged 60+ reported internet use in the past month, with significant geographic disparities (ranging from 40.83% to 88.95%). The prevalence of Alzheimer’s disease among Medicare beneficiaries aged 65+ was 12.92%, with community rates varying from 6.69% to 25.54%. Self-reported cognition difficulty in MA averaged 7.47%, with rates as high as 80.77% in certain communities. In RI, 71.43% of individuals aged 60+ reported recent internet use, with community variations from 54.75% to 88.70%. Alzheimer’s disease prevalence among older adults was 12.04%, with community rates ranging from 7.25% to 16.27%. Cognition difficulty in RI was self-reported by 7.01% of the population aged 65+, with community-specific rates as high as 12.71%. Disparities exist in internet use and cognitive health among older adults. Addressing digital inequalities through targeted interventions can promote cognitive resilience, ensuring equitable access to technology’s benefits for aging populations in Massachusetts and Rhode Island.

